# Alumni Association of the Chicago Medical College

**Published:** 1874-03-15

**Authors:** S. A. McWilliams

**Affiliations:** Secretary


### ALUMNI ASSOCIATION OF THE CHICAGO MEDICAL COLLEGE.

THE Alumni met as usual, in the Lecture-room of the College, on Tuesday, March 10th, at 10 A.M. A larger number than usual were present, and more interest manifested.

The Committee on Prize Essays decided to award the prize of fifty dollars to the author of the essay bearing the motto, "Learn to Labor, and to Wait." On opening the sealed envelope, the name of the author was found to be Geo. H. Fuller, of Class '69, residing at Webster City, Iowa.

The same prizes were offered for another year.

It was moved and carried that Drs. Thos. Bond, Wm. E. Quine, Chas. W. Earle, Lyman Ware, and S. A. Mc Williams, be a committee to provide for a social entertainment, and an interesting programme of exercises for the next annual meeting.

Dr. Bond, the retiring President, delivered a novel, but well-received, address.

It was moved and carried, that the editors of THE MEDICAL EXAMINER be requested to publish the Prize essay, and Necrologist's Report.

It was voted that a sufficient number of copies be published, in pamphlet form, to supply the members, containing the President's Address, Necrologist's Address, and Prize Essay.

The following are the officers for the ensuing year: Dr. D. S. Jenks, Class '66, President (Plano, Ill.); Dr. A. J. Smith, Class '69, Vice-President (Wabash, Ind.); Dr. V. F. Kinney, Class '74, Vice-President; Dr. Wm. E. Quine, Class '69, Necrologist (Chicago, Ill.); Dr. S. A. McWilliams, Class '66, Secretary and Treasurer (Chicago, Ill.)

S. A. McWILLIAMS,
*Secretary.*

